# Effects of 10-MDP Based Primer on Shear Bond Strength between Zirconia and New Experimental Resin Cement

**DOI:** 10.3390/ma13010235

**Published:** 2020-01-05

**Authors:** Francesco Valente, Luan Mavriqi, Tonino Traini

**Affiliations:** 1 Department of Medical, Oral and Biotechnological Sciences, “G. d’Annunzio” University of Chieti-Pescara, 66100 Chieti, Italy; 2 Department of Oral Surgery and Implantology, University “ALDENT” of Tirana, 1023 Tirana, Albania; luanmavriqi@yahoo.com

**Keywords:** zirconia, Y-TZP, 10-MDP, Panavia V5, Surgi Dual Flo’ Zr, resin based luting material, shear bond strength

## Abstract

To date, numerous materials in the dental field are marketed to ensure stable adhesive cementation of zirconia ceramics (Y-TZP). The aims of this study were to assess the shear bond strength of the new experimental cement Surgi Dual Flo’ Zr to Y-TZP compared to Panavia V5 cement, and to evaluate the effect of 10-MDP (10-methacryloyloxydecyl dihydrogen phosphate) containing primer on their bond strength. Twenty composite cylinders and Y-TZP disks were adhesively luted and divided into four groups based on cement type used and application or not of 10-MDP. The groups (n = 5 each) were S 10MDP (Surgi Dual Flo’ Zr with 10-MDP); S no 10MDP (Surgi Dual Flo’ Zr without 10-MDP); P 10MDP (Panavia V5 with 10-MDP); P no 10MDP (Panavia V5 without 10-MDP). Maximum load resistance (ML) and shear bond strength (SBS) were tested and mode of failure qualitative documented via scanning electron microscopy. The data were analyzed with one-way ANOVA, Holm-Sidak method, and Bayesian analysis. ML and SBS were significantly higher in S 10MDP than in S no 10MDP; and in P 10MDP than in P no 10MDP (*p* < 0.05). No significant differences were found between S 10MDP and P 10MDP; S no 10MDP and P no 10MDP (*p* > 0.05). Cohesive, adhesive, and mixed failure occurred among the groups. Bond strength between the experimental resin-based cement and Y-TZP was adequate for clinical application when 10-MDP was added. 10-MDP containing primer was effective improving the bond strength to Y-TZP more than the different type of resinous cement.

## 1. Introduction

The use of zirconia is widespread in dentistry nowadays. One of the most employed is yttria-tetragonal zirconia polycrystal (Y-TZP), which has a broad range of applications in the dental field, in particular regarding fixed prosthodontics [1,2,3]. Y-TZP is superior in terms of mechanical property (strength, toughness, fatigue resistance) to other glass ceramics; however, some inherent problems still remain [4,5,6]. Among others, one problem consists of difficult adhesion to the tooth structures due to its inert surface. Conventional cementation techniques for Y-TZP fixed restorations provide insufficient bond strength [7,8]. Resin bonding is therefore hoped for in various clinical situations, such as when the prepared tooth structure is unusually short or tapered, or when the tooth tissues need to be preserved. Moreover, it is likely that strong chemical adhesion should empower long-term fracture and fatigue resistance of all-ceramic restorations in the oral environment, as well as retention and prevention of microleakage [9]. Zirconia has a crystalline structure, with no glass. Therefore, differing from glass ceramics, acid-etching is not effective in preparing the surface for the adhesive cementation. At the same time, the absence of silica in its structure does not allow suitable direct bonding of composite resin [9,10]. With this intent, several surface conditioning methods are used such as selective etching, laser beam surface etching, grinding, air abrasion with alumina (aluminum oxide, Al_2_O_3_) particles, tribochemical silica (SiO_2_) coating, and application of adhesive/primer, or a combination of them [11].

The results of the use of primers containing 10-methacryloyloxydecyl dihydrogen phosphate (10-MPD) have been encouraging for enhancing the adhesion strength of zirconia to composite resins, generally preceded by alumina sandblasting [12,13]. The chemical linkage between 10-MDP and Y-TZP is a simple and noninvasive to ceramic substrates operation, and it results in excellent bonding outcomes [14,15,16]. Nevertheless, the ideal technique for treatment of zirconia to create an effective chemo-mechanical bond to composite resin is still unknown [17]. Considering the plethora of commercial products available, including primer/adhesives, there is still an unavailability of uniform guidelines for bonding composite resin and resin-based compounds to zirconia ceramics.

Panavia systems (Kuraray Noritake Dental Inc., Okayama, Japan) was considered one of the most effective dual curing resin cements for bonding restorations to dental substrates [18,19,20]. Panavia V5 is the most recent product, provided by the manufacturer since 2015.

A new experimental material (Surgi Dual Flo’ Zr; Miromed s.r.l., Lainate, Milan, Italy) was recently introduced on the market to be used for adhesive cementation of zirconia restorations (specifically Y-TZP) without any primer surface pretreatment. Only sandblasting pretreatment with aluminum oxide particles (Al_2_O_3_) was contemplated.

Following these premises, the authors set up this study with the aim to do the following:

- assess the bond strength of the new experimental cement Surgi Dual Flo’ Zr to Y-TZP compared to Panavia V5 cement;
- evaluate the effect of 10-MDP on the bond strength to Y-TZP of the former cements.

The primary hypothesis being tested was a null hypothesis (*H*_0_) that considered no statistically significant differences in maximum load resistance (ML), and shear bond strength (SBS) for Nano-hybrid composite cylinders cemented onto Y-TZP disks using both Surgi Dual Flo’ Zr with or without 10-MDP and Panavia V5 with or without 10-MDP. A secondary hypothesis (alternative hypothesis, *H*_1_) of significant difference was considered in case of rejection of the (*H*_0_).

## 2. Materials and Methods 

### 2.1. Experimental Resin-Cement (Surgi Dual Flo’ Zr)

The Experimental cement was a dual curing composite based on methacrylate and glass filler with a typical composition summarized in Table 1 and physical properties reported in Table 2. 

### 2.2. Sample Preparation and Experimental Groups’ Identification

The same experienced operator (F.V.) carried out all the steps of the experimental phase. The materials used in the study are listed in Table 3.

**Figure 1:**
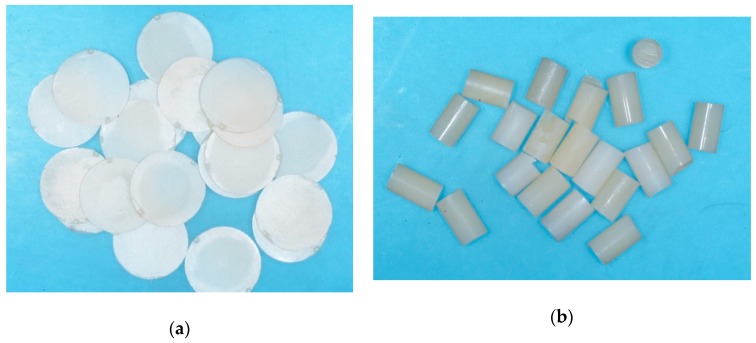
(**a**) Y-TZP disks used for the study (10 mm diameter, 0.5 mm thick) (n = 20); (**b**) Nano-hybrid composite cylinders created for the study (4 mm diameter, 8 mm height) (n = 20).

Twenty Y-TZP disks (10 mm diameter, 0.5 mm thickness) were provided by Miromed s.r.l. (Lainate, Milan, Italy) (Figure 1a). Twenty resin composite cylinders of 4 mm diameter, 8 mm height were prepared using handmade plastic transparent molds (to standardize composite resin size) (Figure 1b). The molds were stuffed with nano-hybrid, light curing resin composite (Enamel Plus UD2; Micerium S.p.a., Avegnano, Genoa, Italy). The composite was light cured with a diode lamp (Valo, 395–480 nm light wavelength, 1400 ± 10% mW/cm^2^ light intensity; Ultradent, South Jordan, UT, USA) for 40 sec at 2 mm distance from four different directions.

Before cementing, the surfaces of the Y-TZP disks were grinded using 600-grit paper under running water to achieve standardized starting conditions. Thereafter, a randomly chosen side surface of each disk to be used for adhesion was sandblasted (Basic Classic, Renfert, Hilzingen, Germany). Operating condition involved the use of 110 μm aluminum oxide particles (Al_2_O_3_) at a pressure of 0.25 MPa with a direction perpendicular to the surface, at a distance of 2 cm for 10 s.

Basing on the adhesion procedures and materials adopted, four different groups of five specimens each (n = 5) were identified:

- S no 10MDP: the composite cylinders were bonded to Y-TZP disks center using Surgi Dual Flo’ Zr cement only;
- S 10MDP: 10-MDP primer (Clearfil Ceramic Primer Plus; Kuraray Noritake Dental Inc., Okayama, Japan) was first applied on the Y-TZP surface by means of a microbrush (Micro Tip Applicator, GC Corp., Tokyo, Japan), let dry at room temperature, then the composite cylinders were bonded to Y-TZP disks center using Surgi Dual Flo’ Zr cement;
- Panavia no 10MDP: the composite cylinders were bonded to Y-TZP center disks using Panavia V5 cement only;
- Panavia 10MDP: 10-MDP primer (Clearfil Ceramic Primer Plus; Kuraray Noritake Dental Inc., Okayama, Japan) was first applied on the Y-TZP surface by means of a microbrush (Micro Tip Applicator, GC Corp., Tokyo, Japan), let dry at room temperature, then the composite cylinders were bonded to Y-TZP disks center using Panavia V5 cement.

One specimen therefore consisted of one composite cylinder adhesively cemented on one zirconia disk. Panavia V5 and Surgi Dual Flo’ Zr cement were mixed and applied with auto-mixing tips provided by the corresponding manufacturers. The exceeding cement was gently removed with a probe and then light cured (with the same lamp used for the curing of the composite cylinders), for 40 s at a 5 mm distance from four directions around the cylinder. The prepared specimens were stored in distilled water at room temperature for one week before testing.

### 2.3. Shear Bond Strength (SBS) Test

**Figure 2:**
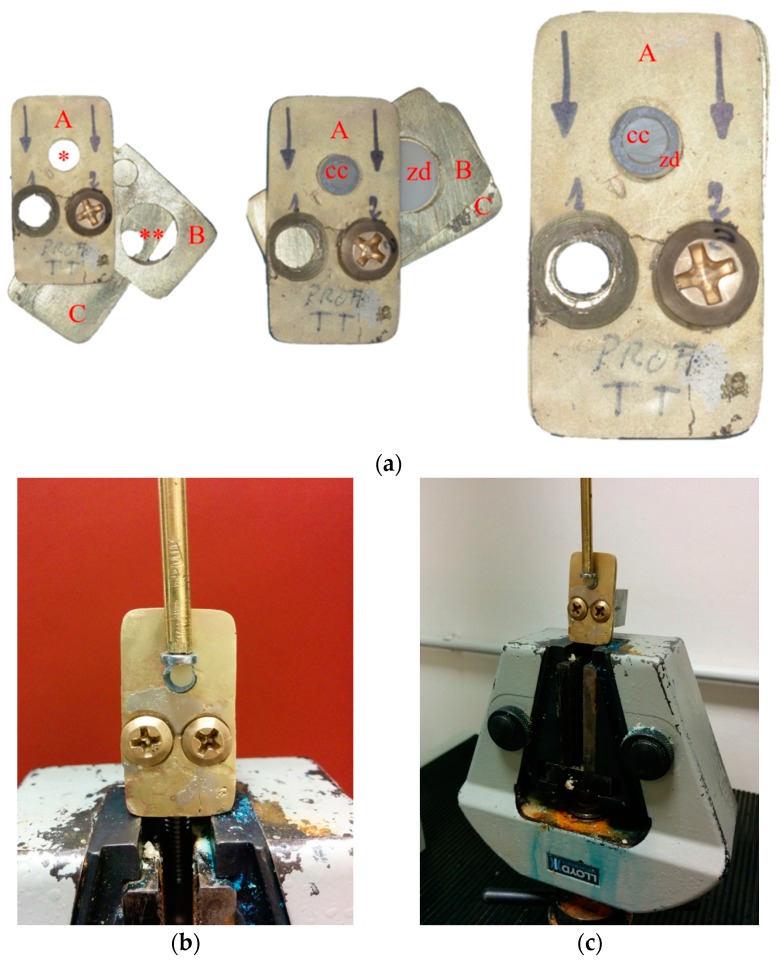
(**a**) The custom-made device in brass used to place the specimens onto the testing machine. It was composed by three assembled plates (A–C) held together by means of two screws (1–2). Plate A was equipped with a hole (*) to fix the composite cylinder (cc). Plate B was equipped with a larger hole (**) to fix the zirconia disk (zd). Black arrows indicate load direction during the testing phase; (**b**,**c**) Broader view of a specimen during the test.

SBS test was carried out with a universal testing machine (Lloyd LR30K; Lloyd Instruments, Ametek STC, Bognor Regis, UK). The specimens were tested one by one, located on the testing machine using a custom-made specimen holder in brass. Description about the specimen holder and its exploitation are shown in Figure 2a–c. To best fit and fix the specimen during the test, and to eliminate risks of damage during mounting, for each specimen the three parts of the holder were disassembled and re-assembled.

**Figure 3:**
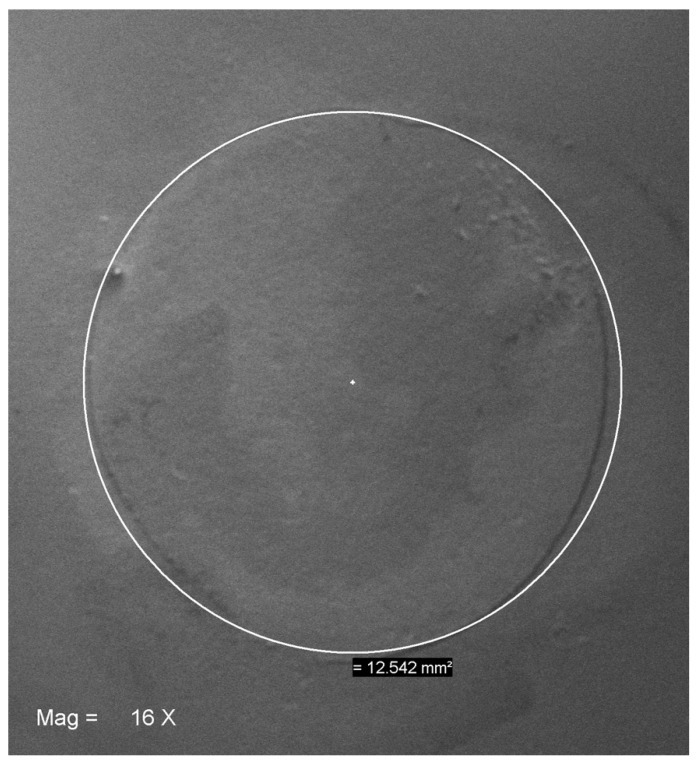
SEM assay of the adhesive cross-sectional area (≅12.54 mm^2^) onto a representative Y-TZP specimen. Mag 16×.

The machine was set in order to have a cross-head speed of 2mm/min until failure occurred (specimen fracture). The corresponding software recorded the maximum load in Newton (N) and maximum stress in MegaPascal (MPa) required to produce a failure. Shear strength (τ) (MPa), was calculated from the standard formula:

τ = *F*_t_/*A* (1)

where *F*_t_ is the applied load in newton (N) and *A* is the shear area in mm^2^. The relative shear area was calculated using the equation of the area for a circle:

*A* = π*r*^2^ (2)

where *A* is the adhesive cross-sectional area (mm^2^) and *r* (mm) is the diameter of the bonded area divided by two, measured using a digital caliper (Mitutoyo Corporation, Tokyo, Japan). It was defined = 12.56 mm^2^. This mathematically derived measure was later assessed by the scanning electron microscopy (SEM) and approximated ≅ 12.54 (Figure 3). 

The modes of failure were defined as cohesive (if the fracture occurred within the mass of the Y-TZP disk or of the composite cylinder), adhesive (if the fracture occurred at the cement/Y-TZP interface), or mixed (a combination of cohesive and adhesive).

Data concerning maximum load until failure (ML, in N) and the shear bond strength (SBS, in MPa) were collected from SBS test.

### 2.4. Scanning Electron Microscopy (SEM) Analysis

After the SBS test, the specimens were prepared for observation under SEM (Zeiss EVO 50 XVP; Carl Zeiss AG, Oberkochen, Germany). The specimens were gold sputtered by means of a sputter coater (Emitech k550; Quorum Technologies Ltd., Laughton, UK), set at 15 mA current intensity, 7 × 10^−2^ mbar of pressure for 2 min, that typically gives 10 nm coating thickness. 

Thereafter, specimens were examined using different magnifications, with the purpose of carrying out a qualitative analysis of failures.

### 2.5. Statistical Analysis 

The data were statistically inferred by means of SPSS 26 statistical software (IBM, Armonk, New York, NY, USA). Both normal distribution (Shapiro-Wilk test) and equal variance (Levine’s test) were used to verify normal distribution of the data; therefore, the one-way analysis of variance (ANOVA) was used to evaluate the overall significance. After that, the post-hoc Holm-Sidak method was used to perform the pair-wise comparisons to identify the P values to reject or not reject the null hypothesis (*H*_0_). The strength of evidence in favor of the alternative hypothesis (*H*_1_) was established using Bayesian analysis to determine the Bayes factor to weigh the evidence hypothesis as posterior probability (*H*_1_) based on the prior probability (*H*_0_). 

*p* < 0.05 was considered the threshold value to detect rejection or significance.

## 3. Results

### 3.1. Maximum load (ML) and Shear Bond Strenght (SBS)

Mean and standard deviation (SD) of ML and SBS for the four groups were listed in Table 4. The data were normally distributed since, normality test (ML *p* = 0.116; SBS *p* = 0.086) and equal variance test (ML *p* = 0.250; SBS *p* = 0.304) passed in both cases for both variables. The highest mean values of ML (68.000 ± 12.083 N) and SBS (4.760 ± 1.258 MPa) were recorded for S 10MDP; S no 10MDP showed the lowest ML (10.580 ± 3.276 N) and SBS (0.840 ± 0.264 MPa) mean values.

**Figure 4:**
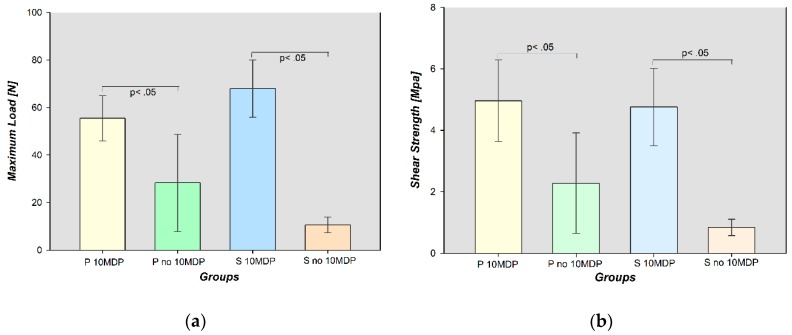
Graphical representation of mean values (±SD) of maximum load and shear bond strength. *p* < 05: statistically significant differences; (**a**) Maximum Load (N); (**b**) Shear Bond Strength (MPa).

One-way ANOVA test and the following Holm-Sidak method for the groups of interest revealed that ML and SBS were significantly higher in S 10MDP than S no 10MDP, and in P 10MDP than P no 10MDP (*p* < 0.05). No statistically significant differences were discovered between S 10MDP and P 10MDP, and between P no10MDP and S no 10MDP, for both ML and SBS (*p* < 0.05). Results are graphically reported in Figure 4a,b. The Power of performed test with alpha = 0.050 was 1.000. 

**Figure 5:**
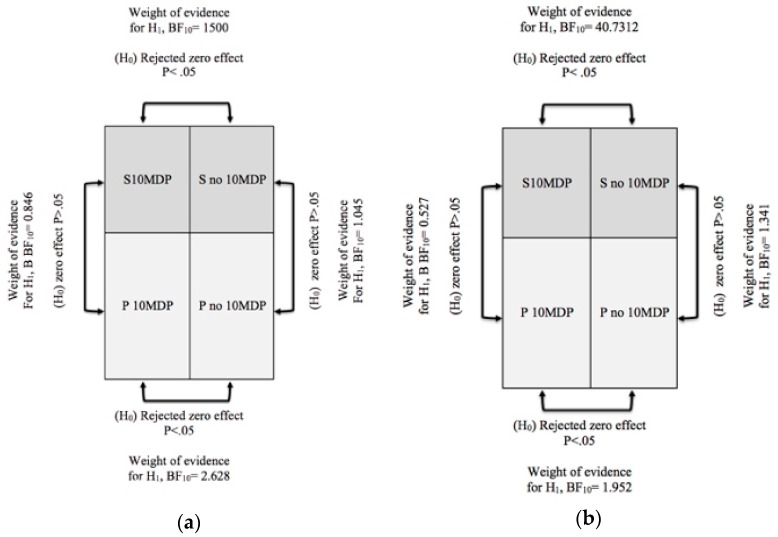
Summary of the results of Bayesian analysis performed for independent samples with Rouder method; BF_10_ = evidence for *H*_0_ and *H*_1_ hypotheses. (**a**) Maximum load test (ML); (**b**) Shear bond strength test (SBS).

Results showed how only the use of resin cements, without 10 MDP, provides low values of ML and SBS even if the Y-TZP surface was sandblasted. The Bayesian analysis with identification of the Bayes factor (BF) provided a tool for inference the weight of the evidence for the *H*_0_ or *H*_1_ hypothesis supported; Figure 5a,b summarize the results reporting the BF.

A different scenario emerged when, after the sandblasting procedure, 10-MDP primer was applied immediately before the resin cement. For Panavia V5 cement there was a statistically significant increase in both ML and SBS mean values (*p* < 0.05) if used without 10-MDP (ML: 28,320 ± 20,418 N; SBS: 2282 ± 1636 MPa) rather than with 10-MDP (ML: 55.500 ± 9.579 N; SBS: 4.960 ± 1.328 MPa). The same was for Surgi Dual Flo’ Zr (ML: 10.580 ± 3.276 N; SBS: 0.840 ± 0.264 MPa without 10-MDP. ML: 68.000 ± 12.083 N; SBS: 4.760 ± 1.258 MPa with 10-MDP) (*p* < 0.05). Nevertheless, the comparison of ML and SBS mean values in these two groups (S 10MDP and P 10MDP) did not reveal statistically significant differences (*p* > 0.05). These results also indicated that comparing the resin cements (without 10-MDP), the new experimental cement resulted in being not significantly different from the Panavia V5 (*p* > 0.05).

### 3.2. SEM Analysis and Modes of Failure

The failure mode analysis revealed that for the specimens of P no 10MDP and S no 10MDP groups failed due to adhesive failure. Otherwise, in the P 10MDP group there was a fracture failure of the zirconia discs (cohesive failure of the Y-TZP). This particular aspect must be considered extremely important since in our experiments we used Y-TZP discs with a thickness (0.5 mm) compatible with the clinical application of the material in ceramic veneers. 

S 10MDP group showed all three kinds of failures (adhesive, cohesive for Y-TZP discs, and mixed). 

**Figure 6:**
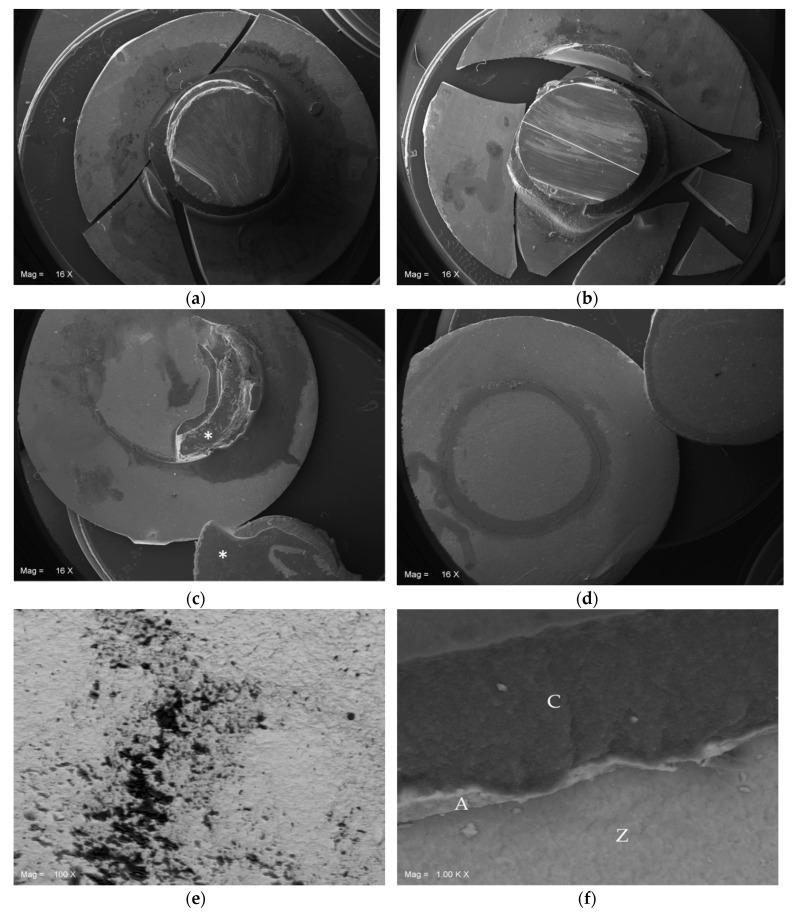
SEM images of representative specimens among groups. (**a**) P 10MDP: cohesive failure within the Y-TZP mass. The resin cylinder remained attached to the center of Y-TZP disk. Mag 16×; (**b**) S 10MDP: cohesive failure within the Y-TZP mass. The resin cylinder remained attached to a fragment of Y-TZP disk. Mag 16×; (**c**) P no 10MDP: adhesive failure. A part of the resin cement mass (*) remained undetached from Y-TZP and resin composite, respectively. Mag 16×; (**d**) S no 10MDP: adhesive failure. The resin cement completely detached from the substrates: the resin cylinder in the upper right corner, and the Y-TZP disk at the center. Mag 16×; (**e**) Particular of Figure 6c: the residual resin cement and 10-MDP containing adhesive (black spots) flood the notches of sandblasted Y-TZP sandblasted surface (grey). Mag 100×; (**f**) Particular of Figure 6c: adhesive interface 10-MDP primer containing (A) comprised between resin cement (C) and Y-TZP (Z). Mag 1000×.

The characteristics of the modes of failure and their particulars can be observed in the SEM images (Figure 6a–f).

## 4. Discussion

The null hypothesis was rejected considering both maximum load resistance and shear bond strength between S 10MDP and S no 10MDP, P 10MDP and P no 10MDP since a statistically significant differences existed. On the contrary, the null hypothesis failed to reject comparing S 10MDP and P 10MDP, P no 10MDP and S no 10MDP, since there were no statistically significant differences.

The results confirm findings of previous studies where the application of phosphate monomer containing primer (MDP and 10-MDP) was effective in bond improving between resin-based luting agents and zirconia ceramics [21,22,23,24,25].

It is evident form Bayesian analysis’ results interpretation that 10-MDP was more effective in increasing ML and SBS of the experimental cement (ML BF_10_ = 1500, SBS BF_10_ = 40.7312) rather than Panavia V5 (ML BF_10_ = 2.678, SBS BF_10_ = 1.952). It can be speculated that some chemo-physical feature of Panavia V5 permits it to have a better bonding performance on sandblasted zirconia than the experimental cement when 10-MDP is not applied. On the other hand, the experimental cement chemical composition could be more receptive towards the 10-MDP molecule. More investigations are necessary to better comprehend this topic.

The 10-MDP molecule consists of a phosphoric-acid group placed at one end of the molecule, a vinyl group at the other end, and a spacer ester chain comprised of ten carbons that separates these two active groups. The vinyl group facilitates polymerization and provides chemical coupling with unsaturated carbon links in the resin matrix of the substrate. The phosphate group is a crucial agent for promoting adhesion with hydroxyapatite or metal oxides such as alumina or zirconia. This is the key group responsible for the 10-MDP chemical bonding ability with non-polar and chemically inert zirconia surfaces [26,27,28,29,30]. In P no 10MDP and S no 10MDP groups, chemical linkage was therefore not present. Only a micromechanical adhesion was provided by the surface roughness, increased by sandblasting, which explains the low mean values of ML and SBS found out. 

In this study, the surface of Y-TZP was airborne abraded with Al_2_O_3_ particles prior to applying 10-MDP aiming to simulate the actual clinical protocol for zirconia adhesive cementation, with the lowest sandblasting pressure and particle size superior to 50 μm to minimize possible surface damage effects and to form an effective micro-retentive surface, respectively [31,32,33]. In order to minimize results affecting variables, during testing phase with the universal machine the point of the applied load was positioned at 2 mm from the adhesion surface (which is the shear area) to be tested. The purpose was to achieve a condition where the secondary effect caused by the bending moment is negligible against the shear strength. Moreover, the authors chose to use composite resin cylinder bonded to zirconia disks so that principally the interface (bonding) area cement/zirconia would have been involved by shear stress. The chemo-mechanical complex cement-composite conceivably ensured a response to loading force as a unique body, promoted by chemical linkage between various acrylate groups in the resin matrix of the two components after polymerization. 

Our results for the P 10MDP group did not reflect findings from previous studies regarding SBS of the product with respect to zirconia ceramics. Reported mean values of SBS are comprised between 31 Mpa and 45 Mpa [24,34]. To the knowledge of the authors, there is a paucity of studies in literature that propose similar experimental conditions to those of the present study, in evaluating Panavia V5 SBS. The variability in the experimental designs (mainly the dimension of the specimens) might be responsible for the difference in findings encountered. Another pivotal answer to this incongruence could be found, in which it was observed that the P 10MDP group showed only cohesive failure, all involving the zirconia disks. On the other hand, the limited thickness (0.5 mm) of the zirconia disks was a clinical compatible choice (resembling the buccal thickness of the ceramic/zirconia veneer crowns). 

In a recent study, Steiner et al. adopted experimental conditions and referred outcomes comparable to the ones of the present study, even though some differences exist, principally in sample processing and in the use of cylinders made of zirconia instead of resin composite. The analyzed cement (Panavia F2.0, with chemo-physical characteristics comparable to Panavia V5) resulted in SBS values of 25.85 ± 11.46 Mpa with 10-MDP primer and 9.18 ± 8.38 Mpa without 10-MDP primer (Clearfil Ceramic Primer Plus) [35].

Results regarding P no 10MDP group are in agreement with the study of Piwowarczyk et al. They evaluated Panavia F without 10-MDP in similar experimental conditions, referred mean values of SBS 2.4 ± 0.4 Mpa not light cured and 6.3 ± 0.8 Mpa light cured [36].

Comparison between S 10MDP and S no 10MDP groups highlights how in S no 10MDP group SBS was not suitable in a clinical context, but in S 10MDP group it reached mean values of the P 10MDP. Therefore, the authors suggest considering revisions in chemical composition, or amendments in the application protocol of the new experimental resin based cement (such as adding 10-MDP based primer), whereby it can be taken into account for clinical application.

This study had limitations. Among the previously stated, the authors did not examine the effect of thermal cycling (simulated aging) on the bonding strength. Therefore, after the advised amendments, further in-vitro studies concerning thermal cycling, a larger number of specimens to test, and various specimen processing are required to assess the thorough bonding potential of Surgi Dual Flo’ Zr.

## 5. Conclusions

Within the limitations of the present in vitro study, the following conclusions can be drawn:

1. Bond strength between the experimental resin-based cement Surgi Dual Flo’ Zr and zirconia ceramic (Y-TZP) is inadequate for its clinical application.
2. Bond strength between the experimental resin-based cement Surgi Dual Flo’ Zr and zirconia ceramic (Y-TZP) significantly increase when 10-MDP is added. Further evaluations are necessary to establish its eligibility for clinical application.
3. Phosphate monomer containing primer (10-MDP) was effective in bond strength, improving between resin-based luting agents and zirconia ceramic (Y-TZP).

## Figures and Tables

**Table 1:** Typical composition (experimental cement).

Component	Value
Barium/silicon dioxide glass	66%
Dental resins based on Bis-GMA	32%
Additives, pigments, catalysts	2%
Total inorganic filler content	66 wt %
Total organic filler content	46 v%

wt %: weight percentage; v%: volume percentage.

**Table 2:** Physical properties (experimental cement).

Property	Value
Compressive strength (after 24 h)	330 MPa
Flexion strength (= transversal strength)	140 MPa
Radio opacity (aluminium)	200%
Linear polymerization shrinkage	0.8%

The measured value (height of the cured polymer cylinder) is divided by two according to ISO 4049; MPa: MegaPascal.

**Table 3:** List of materials used for the experimental phase of study.

Type of Material	Material	Main Composition	Supplier
Zirconia disks	Y-TZP	2–4 mol % Y_2_O_3_ as dopant >98% wt % (ZrO_2_ + HfO_2_) tetragonal zirconia of fine grain size (≅0.2–0.5 mm)	Miromed s.r.l. Lainate, Milan, Italy
Enamel Plus (UD2)	Resin composite	Bis-GMA 1,4-Butandioldimethacrylate UDMA Various silica- based filler Initiators, Catalysts, Accelerators	Micerium S.p.a., Avegnano, Genoa, Italy
Surgi Dual Flo’ Zr	Dual cure resin cement	Refer to Table 1 and Table 2	Miromed s.r.l. Lainate, Milan, Italy
Panavia V5	Dual cure resin cement	A paste: Bis-GMA, TEGDMA, hydrophobic aromatic dimethacrylate, hydrophilic aliphatic dimethacrylate, silanated barium glass filler, fluoroalminosilicate glass filler, colloidal silica, accelerator, initiator B paste: Bis-GMA, hydrophobic aromatic dimethacrylate, hydrophilic aliphatic dimethacrylate, silanated barium glass filler, silanated aluminium oxide filler, accelerator, dl-camphorquinone, pigments	Kuraray Noritake Dental Inc., Okayama, Japan
Clearfil Ceramic Primer Plus	Phosphate monomer—containing primer	3-metacriloxypropyl trimethoxysilane 10-MDP Ethanol	Kuraray Noritake Dental Inc., Okayama, Japan

Y-TZP: yttria-tetragonal zirconia polycrystal; ZrO_2_: zirconium oxide; HfO_2_: hafnium oxide; Y_2_O_3_: yttrium oxide; mol %: mole percentage; wt %: weight percentage; Bis-GMA: Bisphenol A diglycidylmethacrylate; UDMA: Urethane dimethacrylate; TEGDMA: Triethyleneglycol dimethacrylate; 10-MDP: 10-Methacryloyloxydecyl dihydrogen phosphate.

**Table 4:** Mean and standard deviation of the analyzed variables for each group.

Groups	N	ML		SBS	
		Mean	SD	Mean	SD
P 10MDP	5	55.500	9.579	4.960	1.328
P no 10MDP	5	28.320	20.418	2.282	1.636
S 10MDP	5	68.000	12.083	4.760	1.258
S no 10MDP	5	10.580	3.276	0.840	0.264

ML: maximum load (N); SBS: shear bond strength (MPa); SD: standard deviation.
